# Holocarboxylase Synthetase Deficiency: Clinical, Biochemical and Molecular Findings in Five Malaysian Patients Including a Newborn Presenting as Collodion Baby

**DOI:** 10.1002/jmd2.70006

**Published:** 2025-03-06

**Authors:** Siew Li Ting, Yusnita Yakob, Huzaimah Abdullah Sani, Kavitha Rethanavelu, Lock Hock Ngu

**Affiliations:** ^1^ Department of Clinical Genetics Hospital Kuala Lumpur, Ministry of Health Malaysia Kuala Lumpur Malaysia; ^2^ Unit of Molecular Diagnostics Specialised Diagnostics Centre, National Institutes of Health, Ministry of Health Malaysia Kuala Lumpur Malaysia; ^3^ Biochemical Genetics Laboratory, Department of Pathology Hospital Tunku Azizah, Ministry of Health Malaysia Kuala Lumpur Malaysia

**Keywords:** biotin disorder, eczema, encephalopathy, holocarboxylase synthetase deficiency, metabolic acidosis, rashes

## Abstract

Holocarboxylase synthetase (HLCS) is a rare autosomal recessive disorder of biotin metabolism. The mutation spectrum is known to correlate with clinical phenotypes and responsiveness to biotin therapy. Five patients diagnosed with HLCS deficiency between 2015 and 2024 were recruited. Their medical records were retrospectively analyzed for clinical, laboratory, and molecular data. The diagnosis was confirmed through urine organic acid analysis, acylcarnitine profiling of blood spots, and next‐generation sequencing (NGS). All patients had skin rashes, either preceding metabolic decompensation or during follow‐up. Four patients presented in a decompensated state with respiratory distress (100%, 4/4), seizures (50%, 2/4), metabolic acidosis (100%, 4/4), and encephalopathy (100%, 4/4). Most patients (4/5) had late‐onset presentations and responded well to biotin. One patient died before treatment could be given. Of the four who survived, biotin doses of 10–30 mg daily maintained metabolic stability. The oldest patient, now 30 years old, was able to have two successful pregnancies with biotin dose adjustments. Molecular analysis identified 4 mutations: of these, c.1522C>T (p.Arg508Trp) is a known recurrent biotin‐responsive mutation, accounting for 50% of mutant alleles. The c.271del variant had not been previously reported in the literature. This is the first report of HLCS deficiency in a Malaysian population, highlighting the c.1522C>T (p.Arg508Trp) variant as a target for rapid molecular screening. Most patients in this cohort have good outcomes from biotin supplementation, emphasizing the need for early intervention to prevent irreversible neurological damage.

**Summary**

- Holocarboxylase synthetase (HLCS) deficiency can present with symptoms such as metabolic acidosis, developmental delay, seizures, and skin rashes in infancy, though the timing of onset can vary significantly.
- If untreated, it can lead to severe neurological impairments and even death.
- Elevated levels of 3‐hydroxyisovaleric acid in urinary organic acids and 3‐hydroxyisovalerylcarnitine in blood acylcarnitine profiles are sensitive indicators.
- c.1522C>T (p.Arg508Trp) is a known recurrent biotin‐responsive mutation that is common among Malaysian populations.
- Early biotin supplementation is effective in managing symptoms and improving long‐term outcomes when started early before irreversible damage occurs.

## 1 Introduction

Holocarboxylase synthetase deficiency (HLCS; Phenotype MIM number 253270) is an autosomal recessive inherited enzyme defect in the biotin cycle. This enzyme catalyzes the covalent binding of biotin to four biotin‐dependent carboxylases in humans: propionyl‐CoA carboxylase (PCC), pyruvate carboxylase (PC), 3‐methylcrotonyl‐CoA carboxylase (MCC), and acetyl‐CoA carboxylase (ACC), thereby activating them. Defects in HLCS lead to dysfunction of all biotin‐dependent carboxylases, a condition known as multiple carboxylase deficiency (MCD). This leads to impaired gluconeogenesis, fatty acid synthesis, and amino acid catabolism [1, 2, 3].

HLCS deficiency was historically referred to as early‐onset MCD, with patients typically presenting within hours during the neonatal or early infantile period. However, the timing of onset can vary significantly, and even among patients with HLCS deficiency, there exists considerable clinical heterogeneity [3, 4, 5]. Symptoms include acute onset of lethargy and respiratory distress, caused by severe metabolic ketoacidosis and lactic acidosis, along with biochemical findings of characteristic organic aciduria and hyperammonaemia. Other common presentations include seizures, developmental delay, alopecia, skin rashes, and, if left untreated, coma and death may ensue [6, 7, 8]. In late‐onset HLCS deficiency, these recurrent episodes of life‐threatening metabolic decompensation are often precipitated by intercurrent infections and may present at the age of several months to years [8, 9].

HLCS deficiency is considered rarer than the other biotin‐responsive disorder, biotinidase deficiency. The exact incidence of HLCS deficiency is unknown and varies between populations, with an estimated prevalence ranging from 1 in 100,000 live births in Japan [1, 10, 11] to as high as 1 in 10,000 in the Faroe Islands [7]. More than 78 mutations in the *HLCS* gene have been reported in the human gene mutation database (HGMD, available at: http://www.hgmd.cf.ac.uk, accessed October 2024). These mutation spectra have been shown to correlate with the clinical phenotypes. Mutations located within the putative biotin‐binding region demonstrate a good in vivo response to biotin therapy, while mutant enzymes with mutations outside the biotin‐binding site or N‐terminal region show poorer clinical and biochemical responses to therapy [1, 4, 8, 12, 13].

Reports on HLCS deficiency in Southeast Asia are few, and HLCS deficiency in Malaysian populations has not been reported in the literature [1, 6]. We present five Malaysian patients from four unrelated families—two Malays and two Chinese—with a confirmed diagnosis of HLCS deficiency. This case series aims to provide a comprehensive long‐term clinical picture, focusing on their response to biotin, biochemical findings, and genetic spectra. Management of HLCS deficiency during pregnancy is also briefly discussed.

## 2 Methods

All patients with HLCS deficiency who were under follow‐up at our inherited metabolic disease (IMD) clinic at Hospital Kuala Lumpur from 2015 to 2024 were included in the study. Hospital Kuala Lumpur is the main metabolic and genetic referral centre in Malaysia, serving both paediatric and adult patients. Patients' medical records were retrospectively reviewed for clinical features, response to biotin, laboratory studies, and genetic reports. Blood spots for acylcarnitine profiles and urine organic acids are part of the screening tests routinely performed for patients with clinical suspicion of IMD. If these results are suggestive of MCD, biotinidase enzyme activity is measured to distinguish between HLCS deficiency and biotinidase deficiency. The diagnosis is then further confirmed through molecular analysis for biallelic mutations in the *HLCS* gene. Informed consent was obtained from all patients or their legal representatives. As per local regulatory requirements, independent ethics review was not required for case series.

### 2.1 Biochemical Analysis

Urine organic acid analysis is performed using gas chromatography–mass spectrometry (GC–MS). The urine sample is first oximated with methoxyamine hydrochloride, followed by extraction with ethyl acetate, before derivatization with *N*,O‐bis(trimethylsilyl)trifluoroacetamide (BSTFA). Identification of the metabolites was made by comparing the mass spectra of the compounds in the sample with those of the reference compounds in the GC–MS library. The metabolites are semi‐quantitatively reported as small, moderate, or large peaks based on their comparison to the cut‐off values (pseudo units) for each compound.

Blood spots for acylcarnitine were analyzed using electrospray liquid chromatography–tandem mass spectrometry (LC–MS/MS). Reagents from ChromSystems were used for sample preparation, and the underivatized method was employed for analysis. The biotinidase enzyme assay was conducted as a spectrofluorometric assay.

### 2.2 Molecular Genetic Analysis

Patient samples were outsourced to two different laboratories for analysis using next‐generation sequencing (NGS) technology, as this was not available in‐house. Patient 3 passed away without a DNA sample. Whole exome sequencing (WES) was conducted for patients 2, 4, and 5 at 3billion Inc. in South Korea, while an inherited metabolic disease gene panel for patient 1 was outsourced to Invitae in San Francisco, California. Buccal swab samples for patients 1, 2, and 4, and extracted genomic DNA for patient 5 were sent. All samples were collected following the provided protocols, utilizing sample collection kits from the respective laboratories.

All extracted DNA was sequenced according to the manufacturer's protocols using Illumina technology (Illumina, San Diego, CA, USA). The following transcripts for the *HLCS* gene were used: NM_000411.6 (patient 1) and NM_001242784.1 (patients 2 and 4), which share the same nucleotide position, while NM_001352514.2 (patient 5) has a different nucleotide position. The pathogenicity of novel variants was evaluated based on the standards and guidelines established by the American College of Medical Genetics and Genomics (ACMG). Targeted familial variant testing by Sanger sequencing for the parents was subsequently performed locally at the Institute for Medical Research in Kuala Lumpur.

## 3 Results

### 3.1 Clinical Characteristics

A total of five patients with HLCS deficiency (three females and two males) from four families were recruited. Patient 4 was screened and treated from birth due to a significant family history. Of the remaining four patients, one presented early at 1 h of life, while the other three had late‐onset presentations, ranging from 9 months to 24 months of age. The presenting symptoms were consistent with acute metabolic decompensation, including respiratory distress requiring mechanical ventilation (100%, 4/4), seizures (50%, 2/4), metabolic acidosis (100%, 4/4), and encephalopathy (100%, 4/4). All patients exhibited varying degrees of skin lesions that appeared before the age of 1 year. A summary of their clinical characteristics is shown in Table 1.

**TABLE 1:** Clinical characteristics and molecular results of patients.

	P1	P2	P3 (sibling of P4)	P4	P5
Age at last follow‐up	30 years	10 years	Deceased at 2 years	3 years	4 months
Sex	Female	Male	Female	Female	Male
Ethnicity	Malay	Chinese	Chinese	Chinese	Malay
Consanguinity	Yes	No	No	No	Yes
Age at symptom onset	9 months	4 months	9 months	18 months	Within 1 h of life
Symptom at onset	Seborrhoeic dermatitis, metabolic decompensation	Seborrhoeic dermatitis	Dry, erythematous rash, gross motor and speech delay	Eczema	Collodion membrane, metabolic decompensation
Age of 1st metabolic decompensation	9 months	18 months	24 months	Nil	Within 1 h of life
Presentation of decompensation	Respiratory distress, encephalopathy, invasive ventilation	Respiratory distress, encephalopathy, seizures, invasive ventilation, CPR	Respiratory distress, encephalopathy, invasive ventilation	Nil	Respiratory distress, encephalopathy, seizures, invasive ventilation
Age at diagnosis	25 years	18 months	24 months	Detected by newborn screening	Day 3 of life
*HLCS* GENE MUTATION	NM_000411.6^a^	NM_001242784.1		NM_001242784.1	NM_001352514.2
1st allele	**c.1522C>T,p.Arg508Trp**	**c.1522C>T, p.Arg508Trp**	**c.1522C>T,p.Arg508Trp** ^b^	**c.1522C>T,p.Arg508Trp**	**c.2341G>A,p.Asp781Asn**
2nd allele	**c.1522C>T,p.Arg508Trp**	**c.271del, p.Arg91GlyfsTer167**	c.1481G>T,p.Gly494Val	c.1481G>T,p.Gly494Val	**c.2341G>A,p.Asp781Asn**
Current Status	Intellectually normal, married with 2 healthy children	Mild learning disability	Deceased	Normal development	Global developmental delay (coexisting genetic cause of NDD)
Recurrent decompensation	No	No	—	No	No
Rashes	Occasional flare of psoriatic rashes	Resolved	—	Mild eczema at 18 months	Resolved

*Note:* Pathogenic and likely pathogenic variants of the HLCS gene are marked in bold.

Abbreviation: NDD, neurodevelopmental disorder.

^a^ Transcripts of HLCS gene.

^b^ Results presumed to be the same as for sibling (P4).

#### 3.1.1 Patient 1

P1 presented with severe metabolic acidosis following an upper respiratory tract infection. On admission, scaly, erythematous rashes were noted and treated as seborrheic dermatitis. A possible IMD was suspected, and empirical treatment with a combination of vitamins (biotin, B1, B2, B12) resulted in the rapid resolution of acidosis. Initial investigations for an IMD were inconclusive, as the samples were unfortunately lost during transport. Subsequent samples, taken while on supplements, returned normal results. Flare‐ups of skin rashes occurred when she stopped her supplements, resolving upon resumption. Although no formal diagnosis was made at that time, the vitamins were continued due to her remarkable response. She was referred to our clinic as an adult, where she was clinically diagnosed with MCD, and an IMD gene panel confirmed HLCS deficiency.

#### 3.1.2 Patient 2

At 18 months old, P2 presented in a collapsed state following an upper respiratory infection, requiring cardiopulmonary resuscitation (CPR) and high inotropic support. He was encephalopathic and had a brief seizure. The metabolic and lactic acidosis were refractory, and elevated ammonia (146.3 μmol/L) normalized without scavenger therapy. Empirical treatment with thiamine, coenzyme Q10, riboflavin, and protein restriction was initiated. Urgent metabolic tests diagnosed MCD. Further history revealed chronic seborrheic dermatitis since 4 months of age, which was unresponsive to topical steroids. Treatment with oral biotin resulted in dramatic recovery.

#### 3.1.3 P3 and P4

P3 developed erythematous, dry rashes over the periorbital region at 9 months of age, which did not improve with steroid cream or avoidance of common food allergens. Speech and gross motor development were mildly delayed. At 2 years of age, she presented with a fatal metabolic decompensation. Respiratory distress, severe metabolic acidosis, and coma rapidly ensued, leading to her death on day 5 of hospitalization. Investigations for an IMD sent just before her death diagnosed MCD, but it was too late for treatment.

P4, the younger sister of P3, was screened for MCD at 48 h of life due to the family history. She remained metabolically stable and has never been hospitalized.

#### 3.1.4 Patient 5

**FIGURE 1:**
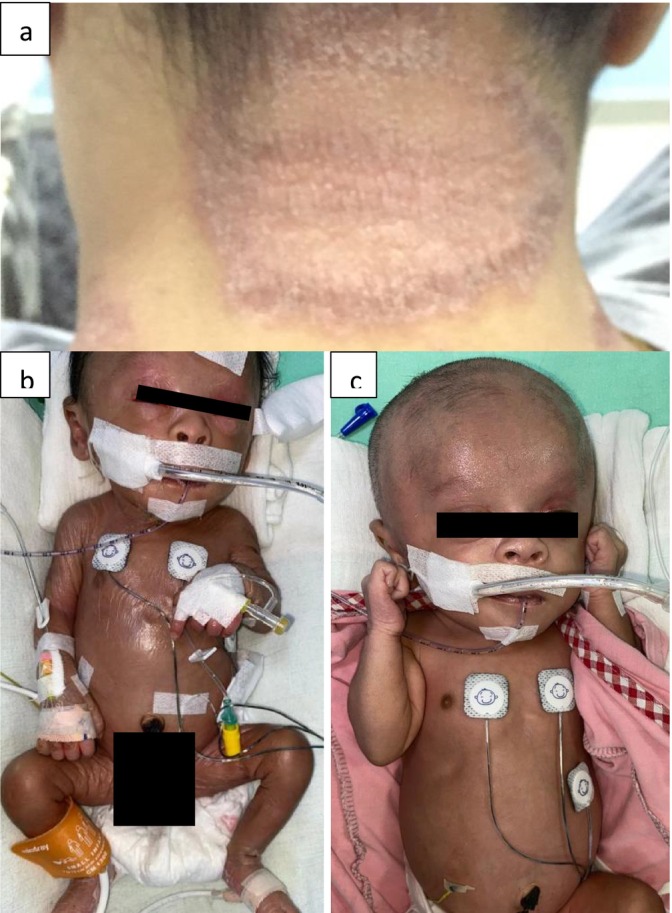
Clinical features. (a) Rashes on the back of the neck of patient 1; (b) and (c) Collodion membrane of patient 5 before and after treatment, respectively.

P5 required resuscitation and intubation within 1 h of life due to respiratory distress and severe metabolic acidosis. Persistent elevated lactate prompted investigations for an IMD, which confirmed MCD. The acidosis rapidly resolved with oral biotin. Seizures developed on day 6 and were controlled with phenobarbital. In addition to his metabolic abnormalities, he is clinically dysmorphic, with multiple congenital anomalies, including a collodion membrane at birth (Figure 1), macrocephaly, ventriculomegaly, laryngomalacia, and abnormal kidneys. His skin improved with biotin and acitretin for presumed autosomal recessive congenital ichthyosis. The family history is significant for a previous child who died during the early infantile period with similar presentations.

### 3.2 Biochemical Results

Metabolites characteristic of MCD include elevated 3‐methylcrotonylglycine (3‐MCG), 3‐hydroxypropionate (3‐HP), 3‐hydroxyvaleric acid (3‐HIVA), and methylcitrate (MeCit) in urine organic acids, as well as elevated levels of 3‐hydroxyisovalerylcarnitine (C5OH) and propionylcarnitine (C3) in the blood acylcarnitine profile [14, 15]. Most patients showed elevations of these metabolites above the cutoff limits at diagnosis, including P4 during newborn screening. Biotinidase enzyme activity was normal for all available patients. Elevated blood lactate levels were present in all, and mild hyperammonaemia was noted in three. Hypoglycaemia was rare, seen only in P3. Most patients continued to show metabolites in their urine after treatment, particularly 3‐HIVA and 3‐MCG. Similarly, C5OH remained above the normal range in half of the patients, although its levels decreased significantly after starting biotin, while C3 levels normalised in all patients. A summary of their laboratory results is shown in Table 2.

**TABLE 2:** Summary of treatment and laboratory data.

	Patient 1	Patient 2	Patient 3	Patient 4	Patient 5
Time of starting Biotin from hospitalization	Day 1	Day 3	Deceased before results available	Day 5	Day 4
Starting dose of Biotin	10 mg OD	10 mg TDS		5 mg BD	5 mg BD
Current dose of Biotin	100 mg TDS	10 mg TDS		10 mg BD	10 mg BD
Investigations at presentation (NBS at 48 h)					
Metabolic acidosis	Yes	Yes	Yes	No	Yes
Lactate (mmol/L)	N/A	16.5	10.5	3.4	13
Glucose (mmol/L)	16	N/A	1.5	4.3	N/A
Ammonia (μmol/L)	11	146	108	92	106
Biotinidase (≥ 77.0 U)^a^	541	259	N/A	123	126
C5OH (0.04–0.47 μmol/L)	N/A	Elevated^b^	26.64	1.76	2.96
C3 (0.22–3.3 μmol/L)	N/A	Elevated^b^	11.11	3.05	8.51
Urine lactate	N/A	+++	++	−	+++
Urine 3‐HIVA	N/A	+++	++	+++	+++
Urine 3‐MCG	N/A	+++	+++	++	+++
Urine 3‐HP	N/A	+++	++	+	++
Urine MeCit	N/A	++	+++	+	Normal
Most recent investigations^c^					
C5OH (0.04–0.47 μmol/L)	0.46	0.58	N/A	0.16	0.78
C3 (0.22–3.3 μmol/L)	1.82	2.40	N/A	1.56	1.89
Urine Lactate	−	+	N/A	+	−
Urine 3‐HIVA	−	+	N/A	+	+++
Urine 3‐MCG	−	−	N/A	−	+++
Urine 3‐HP	−	−	N/A	+	+
Urine MeCit	−	−	N/A	+	++

*Note:* Degree of elevations was designated as follows: +++ = marked increase, ++ = moderate, + = slight, − = not detectable.

Abbreviations: N/A, not available; NBS, newborn screening.

^a^ Normal range in parentheses.

^b^ Where quantitative laboratory reports were not available, results as documented by clinicians in the medical records are used.

^c^ Most recent laboratory investigation were taken during clinic appointments when patients are clinically well.

### 3.3 Response to Treatment and Clinical Outcomes

During long‐term follow‐up, the overall prognosis for patients treated with biotin was good. P3 died before treatment could be initiated, but of the four who received timely treatment, all remained in good health with no further episodes of metabolic decompensation. P1 and P4 are developmentally normal with no neurological sequelae. Patient 2 has mild learning disabilities and attends a special school, but is otherwise independent in activities of daily living. Patient 5 is significantly delayed in all aspects of development but also has multiple congenital anomalies due to a second genetic condition.

P1, currently 30 years old, is the oldest patient in our cohort. She graduated from college with a degree in administration and started a family. Her first pregnancy, unfortunately, ended in a miscarriage at 6 weeks of gestation. During her second pregnancy, severe morning sickness in the first trimester led to a weight loss of almost 8 kg, and she developed worsening rashes on the back of her neck (Figure 1). Urine analysis at this time showed ketosis with mild elevations of 3‐HP and 3‐HIVA compared to her baseline, when well, where there was an absence of metabolites. Biotin was increased to 50 mg twice daily (BD), after which the rashes resolved, and she continued her pregnancy uneventfully. During her third pregnancy, similar rashes in the second trimester improved with an increase of biotin to 100 mg three times a day (TDS). She otherwise remained metabolically stable throughout both pregnancies, even during the surgical stress of delivering her babies by caesarean section. Strict instructions were given to continue her biotin supplementation and maintain glucose‐containing fluids when fasting for the procedures. Blood gas, serum ammonia, lactate, and ketones were monitored during the deliveries and were all normal. Both babies were healthy.

P4 was completely asymptomatic on oral biotin (5 mg BD) until the age of 18 months, when the onset of eczema prompted an increase in the dose to 10 mg BD. A repeat urine analysis showed slight elevations of 3‐MeCit, lactate, 3‐HP, 3‐HIVA, and C5OH from the acylcarnitine profile (0.64 μmol/L). The rashes resolved following the dose adjustment.

### 3.4 Molecular Analysis

NGS identified biallelic mutations in the *HLCS* gene in all patients (Table 1). Although P3 passed away without molecular testing, the mutations and carrier status were confirmed in her younger sister and parents, respectively, making it almost certain that P3 had the same mutations. Consequently, for the purposes of this study, we will presume the molecular results of P3 to be the same as those for P4. Four different mutations were found across 10 alleles: three missense mutations and one frameshift variant. The c.1522C>T (p.Arg508Trp) variant was the most common, occurring in five of the 10 mutant alleles. The other mutations identified were: c.271del (p.Arg91GlyfsTer167), c.2341G>A (p.Asp781Asn) and c.1481G>T (p.Gly494Val). The c.1481G>T variant has been described in a Chinese patient with HLCS deficiency [6], and multiple in silico predictors (PROVEAN, PolyPhen‐2, SIFT, and MutationTaster) suggest that this mutation is likely damaging. For P5, WES revealed dual genetic pathology, with the detection of homozygous likely pathogenic mutations in both the *HLCS* and *GRM7* genes. The latter is associated with an autosomal recessive neurodevelopmental disorder (GRM7; Phenotype MIM number 618922), which causes seizures, hypotonia, and brain abnormalities.

## 4 Discussion

Malaysia has a multiethnic population comprising Malays and indigenous subpopulations (69.4%), Chinese (23.2%), Indians (6.7%), and other ethnicities (0.7%) (https://www.mycensus.gov.my). At our centre, five patients have been diagnosed in the past 9 years. The condition appears to be more common among the Chinese (3/5) compared with other ethnic groups in Malaysia. A previous genomic study in our neighbouring country, Singapore, detected the carrier rate for pathogenic variants in the *HLCS* gene to be 1:831 [16]. It is therefore likely that the actual number of patients is higher, and many may have died undiagnosed.

Majority of our patients presented in critical condition, requiring intensive care support. P4 is the only patient who has never experienced metabolic decompensation, which highlights the success of newborn screening and early initiation of biotin. The metabolites C5OH and urine 3‐HIVA are particularly sensitive, indicating reduced tissue activity of 3‐MCC. P1, the only patient with documented normal urine organic acids when asymptomatic, demonstrated elevated urine 3‐HIVA during her pregnancy. This is likely due to increased biotin requirements in pregnancy. Increased 3‐HIVA excretion rates are an early and sensitive indicator of decreased biotin status in pregnant women, with higher excretion observed during both early and late pregnancy. This suggests that marginal biotin deficiency in late pregnancy is not uncommon [17, 18, 19, 20].

Skin manifestations are the only consistent early symptom in our patients, often presenting months before life‐threatening metabolic decompensation. This should serve as a clue for IMD, particularly if the lesions do not respond to conventional dermatological treatments. However, due to the wide range of skin manifestations, none of our patients were diagnosed based solely on their skin rashes. For P1, P2, P3, and P4, the rashes are described as scaly, erythrodermic, and seborrhoea‐like [21, 22, 23]. The rashes on P1 became psoriatic with age, while P5 had taut, shiny, thickened skin at birth, giving the appearance of a collodion membrane. It is also unclear whether the collodion membrane in P5 can be due to a coexisting autosomal recessive congenital ichthyosis. Given that parents are consanguineous, the possibility of multiple coexisting genetic disorders cannot be ruled out even though WES did not identify mutations linked to this condition. While uncommon, HLCS patients with psoriatic and ichthyosiform rashes have been reported [23, 24, 25].

The missense variants found in our patients (c.1522C>T, c.1481G>T, and c.2341G>A) are located within the biotin‐binding domain. While certain variants are founder mutations in specific ethnic groups, the c.1522C>T mutation has been widely reported across various ethnic backgrounds and is known to be a recurrent mutation [1, 2, 6, 21]. In addition, the c.1522C>T mutation has been shown to produce mutant proteins with higher residual activity, and patients with at least one allele of c.1522C>T did not display symptoms before the age of 2 months [4, 8, 26, 27]. This explains the late‐onset presentations in our cohort and their responsiveness to biotin. P5, who is homozygous for the variant c.2341G>A, is the only patient who presented early, within 1 h of life.

A biotin dose of 10–30 mg per day has been sufficient to prevent further metabolic decompensation in our patients, with adjustments made for age and pregnancy. P1 had remained clinically stable on a dose of 10 mg OD throughout her childhood until pregnancy when a dose of 100 mg TDS was needed to resolve symptoms. This dose is 6000 times higher than the normal intake for non‐affected individuals. There have been no documented adverse effects of high‐dose biotin therapy in pregnant women, with doses up to 600 times the normal intake [28]. A previously reported case described a pregnant patient who had a successful pregnancy and delivery while on a biotin supplement 2000 times the normal intake per day [20, 28]. In contrast, biotin deficiency has been shown to have teratogenic effects in mice [19].

As homozygous null mutations are speculated to be lethal in utero [1, 11], the loss‐of‐function variant c.271del, occurring in compound heterozygosity with c.1522C>T, likely contributed to P2's biotin responsiveness. This variant has, to our knowledge, never been reported in the literature. Although he made an incredible recovery, P2 later demonstrated mild learning disabilities, potentially contributed to by hypoxic injury sustained during CPR. Most patients respond positively to oral biotin supplementation if given before irreversible neurological damage occurs [4, 29, 30].

It is unclear whether increasing the daily biotin dose to normalize mild biochemical abnormalities is necessary in clinically stable patients [8]. At our centre, we do not routinely monitor biochemical markers, as they have been shown to remain persistently abnormal. Rather, dose adjustments are made based on clinical symptoms. Therefore, the optimal biotin dose for each patient must be assessed individually.

In summary, this study contributes to a better understanding of the long‐term clinical outcomes of biotin‐responsive HLCS deficiency in our patients. Due to the significant variability in clinical presentation, diagnosing HLCS deficiency based on clinical findings alone is challenging, and patients may die before diagnostic tests can be performed. Identifying the c.1522C>T hotspot mutation could play a key role in developing targeted molecular assays for the rapid screening of *HLCS* gene mutations in Malaysian patients. The importance of early detection of this highly treatable IEM, before irreversible neurological damage occurs, underscores the critical need for newborn screening.

## Author Contributions

Lock Hock Ngu and Siew Li Ting conceived the work. Siew Li Ting, Kavitha Rethanavelu, Huzaimah Abdul Sani, and Yusnita Yakob collected the data and undertook data analysis and interpretation. Siew Li Ting and Huzaimah Abdul Sani prepared the original draft. Lock Hock Ngu, Siew Li Ting, Huzaimah Abdul Sani, Yusnita Yakob, and Kavitha Rethanavelu critically reviewed the article and revised it for important intellectual content. Lock Hock Ngu supervised the project and contributed to the design and final version of the manuscript. All authors reviewed and approved the final manuscript for submission. Siew Li Ting accepts full responsibility for the work and conduct of the study, has had access to the data, and controlled the decision to publish.

## Ethics Statement

All procedures followed were in accordance with the ethical standards of the responsible committee on human experimentation (institutional and national) and with the Helsinki Declaration of 1975, as revised in 2000. This research was approved and registered in the National Medical Research Register of Malaysia (RSCH ID‐24‐03548‐IEE and NMRR ID‐24‐03990‐NEW). Independent ethics review was not required as per local regulations.

## Consent

Written consent was obtained from the parents or legal guardians and patients older than 16. Additional informed consent was obtained from patient 1 and the parents of patient 5 for the use of their photos. This article does not contain any studies with human or animal subjects performed by any of the authors.

## Conflicts of Interest

The authors declare no conflicts of interest.

## Data Availability

The data presented in this study are available on reasonable and qualified research requests from the corresponding author.
